# Image-guided percutaneous ablative treatments for renal cell carcinoma

**DOI:** 10.1007/s00330-025-11480-w

**Published:** 2025-03-07

**Authors:** Timo A. Auer, Yasmin Uluk, Rosario Francesco Grasso, Roman Kloeckner, Bernhard Gebauer, Thomas Kroencke, Federico Collettini

**Affiliations:** 1 Department of Radiology, Charité—Universitätsmedizin Berlin, Corporate Member of Freie Universität and Humboldt-Universität zu Berlin, Berlin, Germany; 2 Berlin Institute of Health at Charité—Universitätsmedizin Berlin, Berlin, Germany; 3 Department of Radiology, Campus Biomedico University of Rome, Rome, Italy; 4 Institute of Interventional Radiology, University Hospital Schleswig-Holstein-Campus Lübeck, Lübeck, Germany; 5 Department of Diagnostic and Interventional Radiology, University Hospital Augsburg, Augsburg, Germany

**Keywords:** Renal cell carcinoma, Radiofrequency Ablation, Microwave ablation, Cryoablation, Partial nephrectomy

## Abstract

**Abstract:**

In recent decades, percutaneous ablation procedures have evolved into a recognized treatment option for renal cell carcinoma (RCC). Thermal ablation techniques, including radiofrequency ablation (RFA), microwave ablation (MWA), and cryoablation (CA) are now incorporated in most treatment guidelines as a viable alternative, and in some cases, deemed equivalent to nephron-sparing surgery (NSS) or other surgical methods, especially for small renal masses (SRM) up to 4 cm. This review offers an overview of the most prevalent ablation techniques used to treat localized RCC. Additionally, it compares the oncological and clinical outcomes of these techniques with those of surgical options. Finally, it provides an assessment of the role that ablation treatment occupies in current guidelines. In conclusion, the use and incorporation of image-guided minimally invasive treatment options for RCC is on the rise. Existing data suggest that thermal ablation procedures (RFA, MWA, and CA) and partial nephrectomy yield comparable oncologic and clinical outcomes. Despite the data available, the recommendations for thermal ablations vary significantly across national and international guidelines.

**Key Points:**

- ***Question*** *Despite growing evidence and integration in international guidelines, recommendations for ablative procedures in localized small renal cell cancer vary considerably.*
- ***Findings*** *Existing, mostly retrospective, data suggest that thermal ablation and partial nephrectomy yield comparable clinical results for small tumors*.
- ***Clinical relevance*** *Based on the current literature, thermal ablation of renal cell cancer up to 4* c*m in size can be offered to patients as an alternative to surgery*.

## Introduction

Renal cell carcinoma (RCC) is one of the most common malignancies worldwide, according to the Global Cancer Observatory [1]. Each year, more than 300,000 individuals are diagnosed with kidney cancer [2–4]. Recent technical advances in cross-sectional imaging modalities have led to a significant increase in the diagnosis of RCC and especially the detection of small renal masses (SRMs), which are defined as lesions less than 4 cm in size [3]. The strategies available to manage patients with an SRM suspected to be RCC include active surveillance, ablation therapy, and surgery [3]. Most current guidelines prioritize nephron-sparing surgery (NSS), like partial nephrectomy (PN), for SRM management whenever technically feasible [3].

Over the past few decades though, percutaneous ablation procedures have developed into a well-established further treatment option for RCC localized to the kidney. Thermal ablation techniques are now incorporated in most guidelines as a viable alternative or even equivalent to NSS or other surgical approaches [3, 5–8]. The most commonly used ablative procedures include radiofrequency ablation (RFA) and microwave ablation (MWA). As a hypothermal technique, cryoablation (CA) offers some advantages over RFA and MWA including superior visualization of its effect during the procedure and a lower risk of thermal damage at the edges of the ablation zone [9]. This may translate into advantages in the treatment of centrally or near the hilum and the ureter-located SRMs, which generally pose a challenge for NSS. However, based on the current literature MWA, RFA and CA seem to perform equally well in SRMs [10–12].

Aside from thermal procedures, there are other intriguing local ablative treatment options for RCC. Radiation-based techniques, such as Stereotactic ablative body radiotherapy (SABR) and image-guided high-dose-rate (HDR) brachytherapy, are gaining interest in the field of local ablation procedures [13]. Additionally, irreversible electroporation (IRE) is another nonthermal method for treating tumors that are characterized by a low risk to surrounding tissue and critical structures. However, while thermal procedures are widely accessible and straightforward to perform, radiation-based techniques and IRE typically require a high degree of local expertise [14].

Although there is a substantial body of evidence indicating that ablation procedures and surgical resection are similar in terms of outcome and safety in treating small and exophytic renal tumors, national and international guidelines for thermal ablation and treatment protocols vary significantly, and some guidelines do not even provide recommendations for cancers larger than 3 cm or 4 cm [3, 5, 6]. This review provides an overview of the most common ablation techniques for treating localized RCC. In addition, it compares oncological and clinical outcomes with those of surgical options. Finally, we critically review the role assigned to ablation treatment in current guidelines.

## Materials and methods

A thorough review was undertaken by systematically exploring a variety of academic databases, including PubMed, Embase, and the Cochrane Library. This extensive search encompassed a wide range of publications, ensuring the inclusion of relevant studies and data available up until August 2024. This methodical process allowed a robust synthesis of existing knowledge, integrating key findings from diverse sources, and presenting a comprehensive overview of the topic under investigation. Data extraction was meticulously carried out by two independent authors (T.A.A. and Y.U.). In instances where discrepancies arose during extraction, a third author (F.C.) was consulted. This consultative and collaborative approach was implemented to ensure that all data were scrutinized with the utmost rigor. The involvement of a third reviewer to achieve consensus not only strengthened the accuracy and integrity of the extracted data but also bolstered the overall reliability and validity of the findings included in the review. The extracted data are summarized in Tables S1 and S2 in the supplementary material.

## Ablation techniques

### Thermal procedures (Table 1)

#### RFA (Fig. 1)

RFA is the most widely used and accepted ablation technique and was first introduced for RCC in 1997 [15]. The procedure entails inserting one or more radiofrequency electrodes (usually using a multipolar umbrella design) into the tumor tissue using imaging guidance [16]. These electrodes work by delivering an electrical current that induces ionic agitation within the tumor tissue. This process generates heat, raising the temperature of the targeted area to above 60 °C. At such high temperatures, the heat causes proteins and cellular structures to denature, leading to cell death through a process known as coagulative necrosis. Allowing for different device-specific protocols, a common technique for RFA typically begins with an initial electrical power setting of 30–40 W. The power is then gradually increased at a rate of 10 W/min. Power delivery is interrupted briefly twice, known as breaks or roll-offs, to ensure optimal energy delivery and tissue destruction while minimizing the risk of overheating or damage to surrounding tissues [16–18]. Research has demonstrated that RFA offers excellent overall survival (OS), 5-year cancer-specific (CSS), local recurrence-free survival (LRFS), and metastasis-free survival (MFS) rates of 75.8%, 97.9%, 93.5%, and 87.7%, in lesions with a mean size of 2.9 cm, respectively [19].

**Table 1:** Summarizes the different ablations techniques, their principles, advantages and disadvantages, and the selected important literature

Ablation technique	Principle	Advantages	Disadvantages	Important study data
RFA	Heat (radiofrequency)-induced coagulation necrosis	Short procedural time Low costs Low risk of bleeding	No real-time visualization Restricted to lesion size Potentially painful “Heat-sink effect”	- Wah et al [19]: OS, 5-year CSS, LRFS, and MFS rates of 75.8%, 97.9%, 93.5%, and 87.7%, in lesions with a mean size of 2.9 cm. - Andrews et al (2019); *n* = 1798 (PN; RFA; CA); 5-year CSS for RFA: 96%.
MWA	Heat (electromagnetic waves)-induced coagulation necrosis	Short procedural time Low risk of bleeding Reduced “Heat-sink effect”	No real time visualization Restricted to lesion size Potentially painful	- McCloskey et al (2024): 89% of patients followed up remained cancer-free, some at 7 years FU - Chlorogiannis et al (2024): At 8-year follow-up, the estimated survival rates for the MWA cohort were 98% for OS, 97% for RFS, and 97% for MFS. No significant difference vs RAPN.
CA	Freeze (liquid-nitrogen based)-induced necrosis + chemical effects	Real time visualization Lesion size up to 10 cm Less painful	Longer procedural time Multi-probe procedure High costs	- Bhagavatula et al (2020): DSS of 88% (15-years FU) - Chan et al (2022), including RFA and CA: on univariate analysis, all oncological outcomes were comparable amongst CRYO, RFA, and LPN (*p* > 0.05). Patients undergoing CRYO and RFA had a significantly smaller median decrease in eGFR post-operatively compared to LPN (T1a: *p* < 0.001; T1b: *p* = 0.047).
SABR	Radiation (teletherapy)-induced necrosis	Non-invasive No “Heat-sink effect” Less risk for critical structures	Limited data	- Siva et al (2022): 100% CSS survival and no local failures at 43-month follow-up; 10% experiencing grade ≥ 3 toxicity and 74% grades 1–2 toxicity.
HDR brachytherapy	Radiation (brachytherapy)-induced necrosis	No “Heat-sink effect” Less risk for critical structures Single fraction	Limited data	- Damm et al (2019): investigated 16 patients with RCC. The local control rate was 95% including repeated HDR-BT of two recurrences.
IRE	Electrical pulses to disrupt cell membranes	Non-thermal No “Heat-sink effect” Less risk for critical structures	Limited data Complex probe positioning	- Wah et al [39]: CT-guided IRE of clinical T1a (cT1a) RCCs; Technical success rate was 73.3%. he overall 2- and 3-year cancer-free, local-recurrence-free and MFS rates are 89%, 96%, 91%, and 87%.
Histotripsy	Focused ultrasound to destroy tissue	Non-invasive No “Heat-sink effect” Less risk for critical structures	Limited data	Ongoing trials: CAIN trial (NCT05432232) #Hope4Kidney

A comprehensive overview of the literature is provided in Tables S1 and S2

**Fig. 1:**
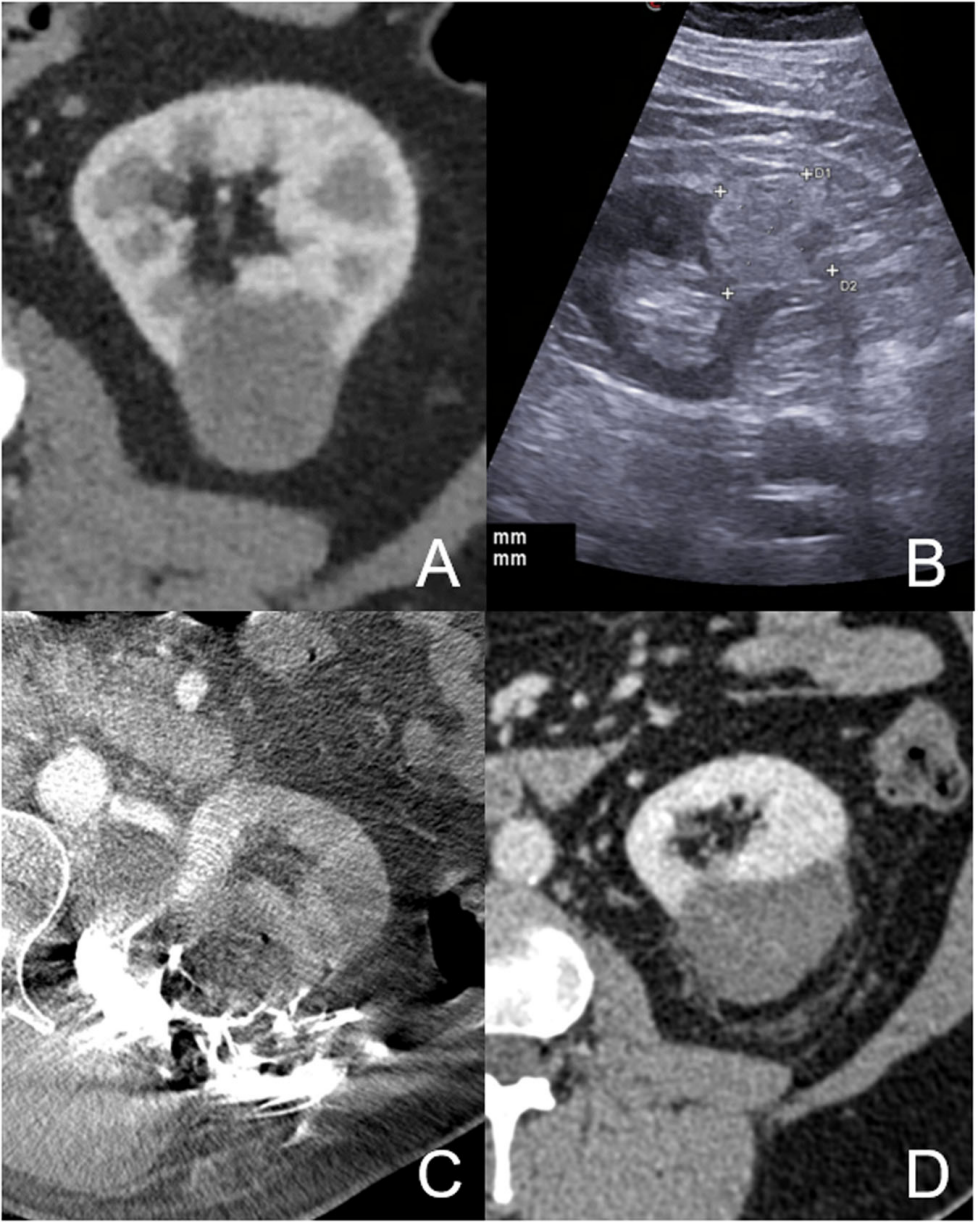
**A**, **B** Shows the contrast-enhanced CT in the weeks before the ablation and the native sonogram of a 36-mm papillary RCC at the lower pole of the left kidney. **C** Shows the intraprocedural cone-beam CT during RFA with ablation for 12 min and hydrodissection. **D** Shows the CT as a control scan with good local tumor control

The primary limitations of RFA are due to the inherent physics of the procedure. One key issue is the dissipation of heat to nearby blood vessels, known as the “heat-sink effect”, which can alter the shape and size of the ablation zone. As a result, treating lesions that are 3 cm or larger becomes challenging. Furthermore, this effect also prolongs the treatment time, potentially increasing the risk of complications. Additionally, RFA typically creates spherical ablation zones, which may not be ideal for all tumor shapes. Another limitation of RFA is poorer intraprocedural visualization compared with other techniques such as CA. Additionally, the proximity of risk structures, such as the bowel, further restricts the procedure’s applicability [16, 17, 20].

#### MWA (Fig. 2)

Like RFA, MWA is a heat-based procedure and has a similar effect grade; however, MWA has been available as an interventional ablation method for a significantly shorter period, having been introduced in 2008 [21]. MWA uses electromagnetic waves to produce heat and cause cell death through hyperthermal injury. A needle-like probe, or antenna, is inserted into the tumor, emitting microwave energy to create an electromagnetic field. This field generates frictional heating, raising temperatures to over 100 °C [17, 22]. MWA is particularly effective in heating larger tumor volumes because, unlike RFA, where the active heating zone is confined to a few millimeters around the electrode, MWA can heat tissues up to 2 cm away from the antenna [23, 24]. While, as a thermal method, MWA faces similar limitations as RFA (proximity of risk structure), its heat-sink effect is generally considered to be less of an issue because of the higher and faster energy transfer of MWA. Nevertheless, these higher energy levels and temperatures theoretically increase the risk of complications and damage to surrounding structures, such as vessels [17, 23, 24]. Nonetheless, similar limitations regarding size exist, and the results presented in the literature are therefore comparable. MWA has reported outcomes of survival and recurrence-free survival (RFS) rates of up to 97–98%, comparable to the outcomes of PN in T1a and T1b tumors up to 7 cm [25, 26].

**Fig. 2:**
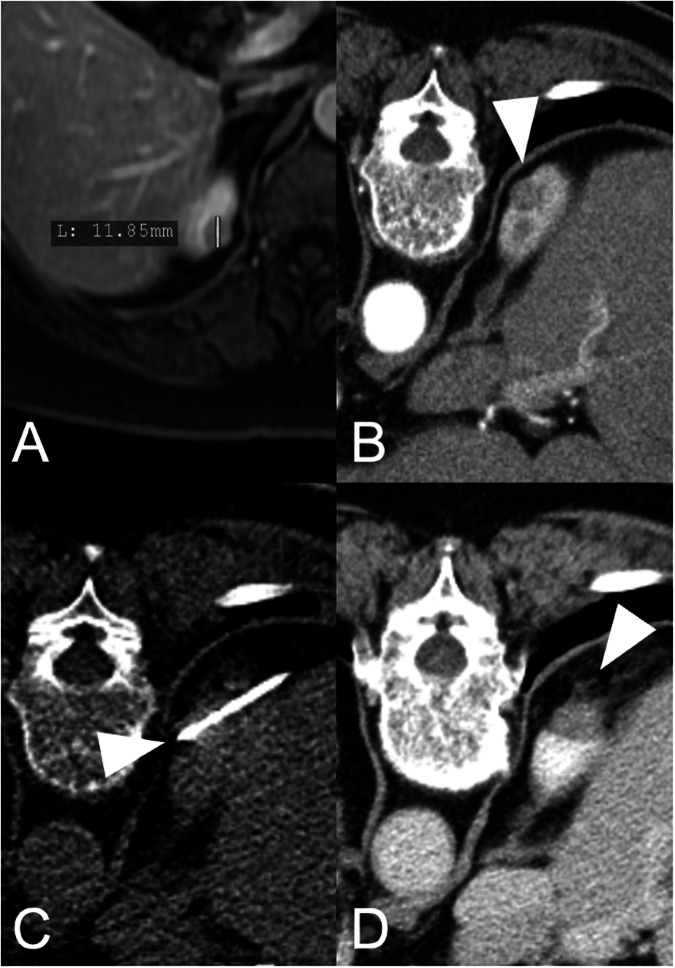
Seventy-nine-year-old female patient who presented for a second opinion. Initially, the patient was offered a resection of the upper third of the kidney. **A** Pre-interventional axial MRI slice with a 12 mm exophytic lesion suspicious for RCC in the upper pole of the kidney. Due to its size and location, the patient was offered MWA and diagnostic puncture for histologic confirmation in one session in analgosedation (fentanyl and midazolam). **B** Planning scan in the prone position and arterial phase (lesion indicated by white arrowhead). **C** Peri-interventional fluoroscopy CT scan with the position of the MWA antenna in the center of the lesion (white arrowhead). **D** Postinterventional scan obtained directly after ablation, indicating sufficient coverage of the lesion by ablation. The white arrowhead is pointing at the post-ablation area. Histology confirmed the diagnosis of clear cell RCC

#### CA (Table 2 and Figs. 3, and 4)

CA is a technique that utilizes argon or liquid nitrogen-based cryoprobes to reduce the temperature within the tumor to below −40 °C. The procedure generally involves two freeze-thaw cycles, typically consisting of 10 min of freezing followed by variable times of active and passive thawing. However, different freeze-thaw cycles have been proposed [27, 28]. The key advantage of CA is its outstanding periprocedural visualization. The ice ball that forms around the needles is easily visible on both CT and MRI scans, and to a lesser extent also on ultrasound. Scans acquired during the freezing cycles can help monitor and control the size of the ice ball, and therefore the ablation zone. Additionally, using multiple needles enables the creation of ablation zones of various shapes and sizes leading to the ability to treat larger tumors, including T1b lesions. Although CA is considered a relatively new thermal procedure, reliable long-term experience has been gained by some centers [29, 30]. Bhagavatula et al reported intermediate- to long-term outcomes in 307 patients with a 10-year and 15-year disease-specific survival (DSS) of 88% [30].

**Table 2:** Overview of the current guidelines on the management of RCC and the role of local ablation techniques

Guidelines	Role of ablation techniques	Remarks
German National Practice Guideline (S3)	Recommend TA as an option only for patients with renal cancers < 4 cm and with significant comorbidities, a limited life expectancy, or contraindications to general anesthesia Recommend surgery whenever feasible	No recommendation for T1b tumors
European Association of Urology (EAU)	No significant difference in overall complication rates between PN and CA Patients with lesions up to 4 cm treated with TA had a shorter average length of hospital stay Panel concluded that the current data were inadequate to reach conclusions regarding the clinical effectiveness of CA vs PN Given these uncertainties regarding the guideline panel decided to recommend CA can only for frail and/or comorbid patients with SRMs	For T1b lesions, local tumor control rates drop significantly
European Society of Oncology (ESMO)	TA and SBRT are non-surgical options, particularly in patients with small cortical tumors Especially appropriate for patients who are frail, present a high surgical risk, have a solitary kidney, compromised renal function, hereditary RCC or multiple bilateral tumors	Recommendations include T1 tumors up to 7 cm, thus T1a and b
National Comprehensive Cancer Network (NCCN)	Ablative techniques are among the primary treatments for T1a tumors	Ablative techniques are not among the primary treatments for T1b tumors
American Urology Association (AUA)	Clinicians should prioritize PN for SRM management when intervention is indicated clinicians should consider thermal ablation as an alternate approach in all patients with tumors < 3 cm in size, where oncologic outcomes are comparable for thermal ablation and PN	Prioritized for lesions up to 3 cm

**Fig. 3:**
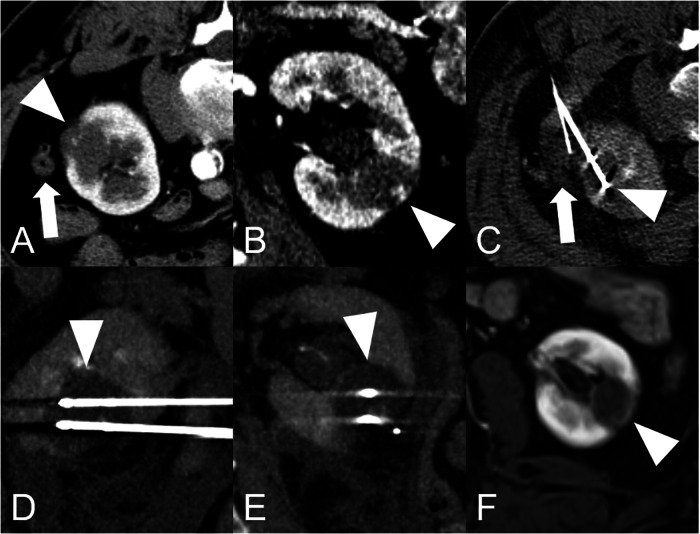
Sixty-four-year-old patient with suspected RCC. **A** (axial), **B** (coronar) Lesion measuring approx. 2.2 cm in the middle third of the kidney (white arrowhead) with an exophytic portion and growth towards the hilus. As the bowel is also closed (white arrow), the decision was made to perform CA (in analgosedation) with hydrodissection of the bowel. **C** CT-fluoroscopy image after 2 min, at the beginning of the 1st freezing cycle. The cranial of two needles is in the middle of the lesion (white arrowhead), while the white arrow is pointing at the fluid collection. The bowel was easily mobilized using 22 G needles and NaCl. **D**, **E** Control scan obtained after 5 min, in the second freezing cycle. The white arrowheads point to the clearly visible ice ball, and the parallel needle positions and the geometric shape of the visible ice ball can be seen in the sagittal and coronal slice guides angled onto the needles. **F** Complete ablation in the control area scan after 6 months in the MRI (T1 contrast-enhanced fat sat sequence). The histologic specimen yielded the diagnosis of papillary RCC

**Fig. 4:**
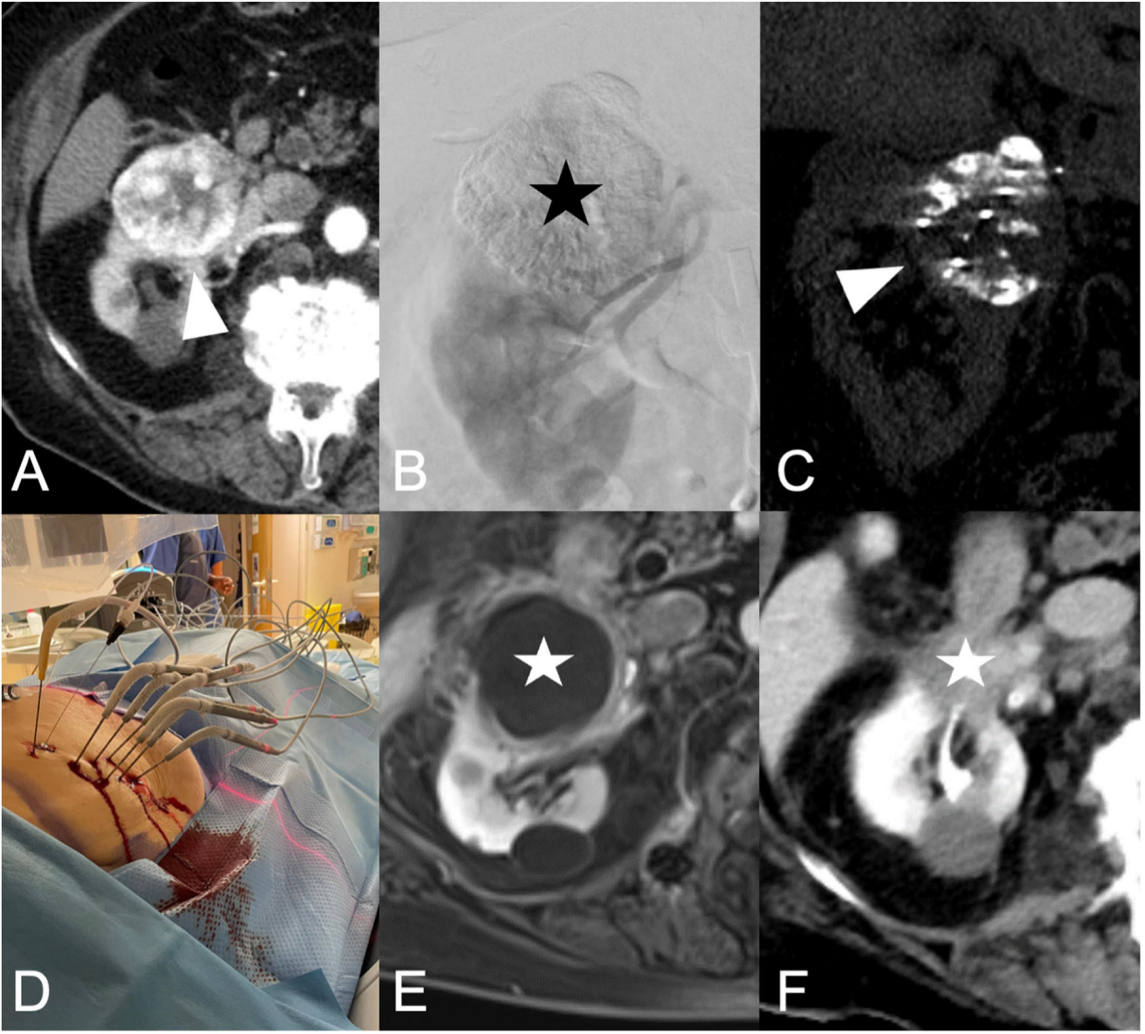
Eighty-eight-year-old female patient with a strongly arterially hypervascularized RCC measuring 6 cm (white arrowhead) and the duodenum as an adjacent risk structure (**A**). Since the patient was in good general health and declined anesthesia, a CA was chosen. Due to the significant arterial hypervascularization, the lesion was angiographically transarterially embolized the day before the ablation (with alcohol and lipiodol). **B** Final DSA (digital subtraction angiography) and a strong lipiodol deposition in the tumor (black asterisk), while the kidney is well perfused. **C** Shows the control scan after 5 min, in the first freeze cycle. The strong lipiodol deposition in the tumor impairs visualization of the ice ball and the seven needles positioned for treatment. To the right of the tumor, there is a small 22 G needle for hydrodissection. **D** Intraprocedural image obtained during the freeze cycle. The patient underwent the procedure without significant pain and remained hemodynamically stable. **E** Control MRI was performed after 3 months, demonstrating complete devascularization of the lesion (white asterisk). **F** Scar formation 8 months after the ablation (white asterisk). Histology of the sample taken at the beginning of the intervention confirmed the diagnosis of clear cell RCC

### Radiation-based procedures

Radiation-based treatments for localized RCC are not very common or well-established. This is partly because effective local ablative alternatives are available, and also because these techniques are typically only accessible at larger hospitals or specialized clinics. However, similar to the trend seen with HCC, radiation-based procedures are gaining increasing significance [31].

#### Stereotactic body radiotherapy

SABR is an innovative, noninvasive curative treatment for patients with primary renal cell cancer. Unlike thermal ablation, SABR is suitable for both T1a and T1b or larger tumors. [13]. In 2022, Siva et al concluded through meta-analyses that SABR is a safe and effective long-term treatment for patients with primary RCC. While single-fraction SABR may result in lower local failure rates compared to multifractional treatments, additional evidence from randomized trials is needed to determine the optimal treatment regimen. These mature findings further support the use of renal SABR as a treatment option for patients who are either unwilling or unfit for surgery [32]. The abovementioned data was already incorporated into the latest guideline of the European Society of Medical Oncology (ESMO) [7]. In a recent phase 2 trial also published by Siva et al SABR demonstrated a 100% CSS survival and no local failures at 43-month follow-up. However, adverse events were notable, with 10% experiencing grade ≥ 3 toxicity and 74% grade 1–2 toxicity [33]. Another phase 2 trial reported a significant decline in renal function post-SABR. Additionally, biopsies performed one year after treatment occasionally revealed viable tumor cells, raising questions about long-term efficacy [34].

#### CT-guided HDR brachytherapy (Fig. 5)

CT-guided HDR brachytherapy is an interstitial radioablative technique that involves temporarily placing an iridium-192 source into the target lesion through a catheter utilizing image guidance. The effectiveness of CT-guided HDR brachytherapy is not affected by the cooling effect of nearby large vessels, and the size of the tumor does not pose a limitation [31]. Moreover, structures like the ureter are not considered at risk, while rapidly dividing cell structures, such as the intestinal mucosa, are. As the treatment does not take place in the CT but in the radiation room, hydrodissection is not possible. Despite this, brachytherapy remains a highly controllable ablation technique. However, published data on HDR brachytherapy for RCC are scarce and limited to individual studies [35].

**Fig. 5:**
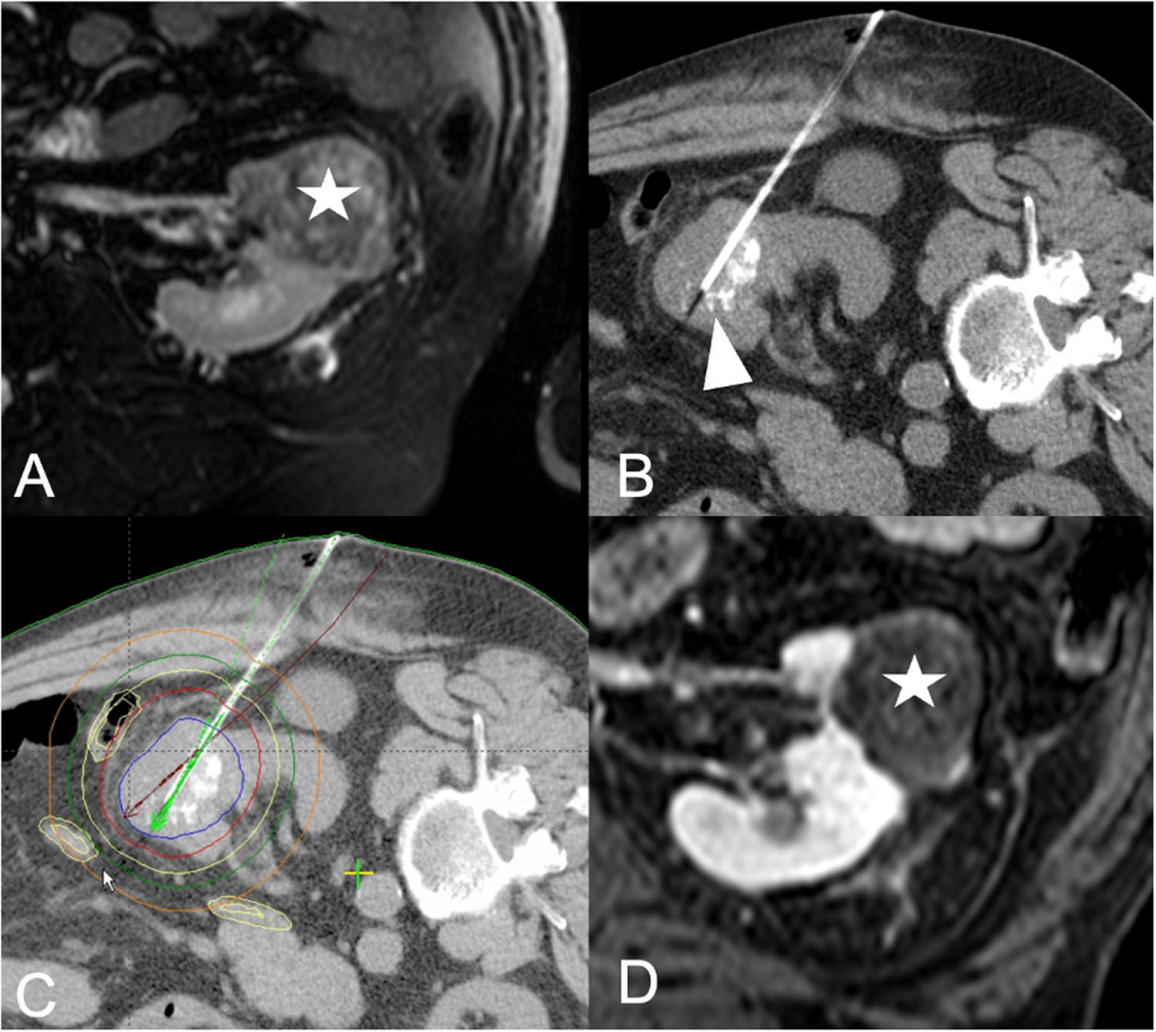
Seventy-one-year-old patient with a histologically confirmed clear cell RCC after prior treatment by transarterial embolization the day before the ablation. **A** The 4.1 cm lesion is located in the central anterior third of the kidney (white asterisk). The patient was referred for CT-guided HDR brachytherapy in analgosedation. **B** Intraprocedural control scan and one of three brachytherapy catheters within the lesion (white arrowhead). **C** Radiation plan. The blue line encircles the complete tumor volume with the surrounding red line indicating the 20 Gy isodose line. **D** Control MRI scan obtained after 6 months, demonstrating complete ablation with local tumor control

### Non-thermal non-radiation-based procedure

#### IRE

IRE, is a nonthermal ablation technique used for local cancer treatment. Unlike traditional thermal ablation methods, which use heat or cold to destroy cancerous cells, IRE relies on electrical pulses to disrupt cell membranes. This disruption causes permanent damage to the cells, leading to cell death without significantly affecting surrounding healthy tissues. The collagen-sparing properties of IRE allow the preservation of vital structures like the ureter, collecting system, and vessels [36]. The main advantage of IRE compared to thermal methods is that it does not rely on high temperatures, which minimizes the risk of collateral damage. As a result, IRE is regarded as the safest option for treating cancers located close to large vessels [37]. One drawback is the need for symmetrical needle placement, which can occasionally be challenging for the interventionalist. Although IRE is a cutting-edge technique, long-term data are already available for specific organs, like the prostate [38]. Wah et al evaluated the safety and efficacy of CT-guided IRE of clinical T1a (cT1a) RCCs close to vital structures and to assess factors that may influence the technical success and early oncological durability. The technical success rate was 73.3%. he overall 2- and 3-year cancer-free, local-recurrence-free, and MFS rates are 89%, 96%, 91% and 87%. The authors concluded that CT-guided IRE in cT1a RCC is safe with acceptable complications. Notably seven residual diseases were successfully ablated with CA, achieving an overall technical success rate of 97% [39]. Overall, data for IRE in RCC is still limited but the technique represents a problem-solving option in critical cases [17].

#### Histotripsy

Histotripsy is a type of therapeutic-focused ultrasound designed to destroy targeted tumors and cancerous tissue—initially focused on liver tumors—without requiring invasive incisions, needle penetration, or thermal intervention. This novel, non-thermal, ultrasound-based technique induces mechanical cavitation, leading to precise cellular destruction. The ongoing CAIN trial (NCT05432232) and the #HOPE4Kidney trial are investigating its potential, suggesting that histotripsy could become a transformative advancement in RCC management.

Table 1 summarizes the different ablations techniques, their principles, advantages and disadvantages, and the most important literature (Table 1).

### Comparative oncologic outcomes

The classical thermal ablation methods (RFA, MWA, and CA) yield effective and safe outcomes in patients with SRM. While some authors highlight the benefits of MWA regarding tumor control, other authors favor CA due to factors like improved intraprocedural visualization. Nevertheless, particularly concerning safety, there are currently no well-structured studies that demonstrate a benefit for a technique in lesions smaller than 3 cm [9, 10, 19, 40]. In SRM management, the key consideration is not finding a superior option among the different thermal ablation techniques, but rather comparing them to surgical approaches like PN in order to identify subgroups of patients who will benefit from either PN or ablation. Despite promising results, thermal ablation techniques are only mentioned by some guidelines for the treatment of T1b stage RCCs [3, 27, 28]. Although especially effective when combined with transarterial embolization, reports in the literature support CA for RCCs up to 10 cm in size [41–43]. Nevertheless, the risk of complications and recurrences rises with lesion size. Therefore, thermal ablation methods should only be considered as an alternative after surgery in lesions < 4 cm [5, 41, 42].

Initial data on SRMs reveal no significant differences between thermal ablation techniques and PN but reliable long-term data is still scarce. In their study of 2022, Chan et al evaluated the long-term outcomes of image-guided ablation and PN for T1 tumors, concluding both procedures to be equally effective while highlighting the positive impact on kidney function when ablation techniques are used [44]. Chlorogiannis et al retrospectively compared long-term oncologic outcomes of CT-guided MWA and robot-assisted partial nephrectomy (RAPN) in patients with T1 RCC [26]. After propensity score matching, 71 patients underwent percutaneous MWA and 71 underwent RAPN. At 8-year follow-up, the estimated survival rates for the MWA subgroup were 98% for OS, 97% for RFS, and 97% for MFS. The matched subgroup that underwent RAPN demonstrated survival rates of 100% for OS, 98% for RFS, and 98% for MFS. Log-rank testing revealed no significant differences between these rates, with p-values of 0.44, 0.67, and 0.67, respectively. Similar to Chan et al, the authors concluded that MWA and RAPN are equally effective in terms of oncologic outcome [26, 44]. Interestingly and contradictory to current guidelines, the study populations investigated by both Chan et al and Chlorogiannis et al included a relevant portion of T1b RCCs up to 7 cm [3, 5, 6, 26, 44].

The study findings of Chan et al indicate that the oncologic outcomes of the classical thermal ablation techniques and PN appear to be comparable [44]. However, beyond oncologic results, considering factors such as complications and the impact on kidney function, it is important to question, from a scientific perspective, whether the patient groups compared are truly equivalent. There is a significant lack of high-quality evidence regarding the management of SRMs, as previous traditional randomized controlled trials were unable to achieve their target enrollment numbers [45]. Neves and colleagues investigated the feasibility of recruitment to a cohort-embedded RCT comparing CA and robotic partial nephrectomy (RPN). A total of 200 participants were recruited to the cohort, of whom 50 were enrolled in the RCT. In the CA intervention arm, 84% of patients consented and 76% underwent CA; in the control arm, 100% of patients underwent RPN. The retention rate was 90% at 6 months. In the RPN group, 2/25 (8%) were intraoperatively converted to radical nephrectomy. Postoperative complications (Clavien–Dindo grades 1–2) occurred in 12% of the CA group and 29% of the RPN group. The median length of hospital stay was shorter for CA (1 vs 2 d; *p* = 0.019). At six months, the mean change in renal function was −5.0 mL/min/1.73 m^2^ after CA and −5.8 mL/min/1.73 m^2^ after RPN [45]. The authors concluded that this feasibility study met its primary endpoint and demonstrated the feasibility of recruitment to an open-label RCT [45]. The data strongly emphasize the comparability of ablative techniques, with a particular focus on CA, when measured against contemporary renal parenchyma-sparing surgical procedures. This evidence highlights that CA, as an ablative method, achieves clinical outcomes that are closely aligned with those of state-of-the-art nephron-sparing surgeries, thereby corroborating its potential as a viable minimally invasive alternative. The findings suggest that CA, alongside other ablative modalities, offers a comparable efficacy in preserving renal function while maintaining oncologic control, thus positioning it as a valuable treatment option within the spectrum of renal preservation strategies [45, 46]. Alongside this data, Abu-Ghanem et al concluded in a systematic review of the European Association of Urology Renal Cell Cancer Guideline Panel that TA could cautiously be offered as an option due to many remaining uncertainties regarding its effectiveness [47]. Another innovation in both increasing the oncological precision of tumor ablation and reducing complications is the technique of stereotactic RFA. However, to date, only larger data on the ablation of liver tumors and only case studies on kidney tumors exist [48].

### Complications

Regarding complication rates and adverse events, Junker et al prospectively compared complications and readmissions after PN and percutaneous CA of T1 RCCs [49]. The study population included 86 partial nephrectomies and 104 CA. The complication rate within 90 days was 23% after PN and CA (*p* = 0.98), with major complication rates of 3% after PN and 10% after CA (*p* = 0.15). The readmission rates were 14% and 11% after PN and CA, respectively (*p* = 0.48) [49]. The authors conclude that PN and CA are comparable regarding complications within 90 days after treatment. Another more comprehensive overview offers a systematic review and meta-analysis from Deng et al [50] including 17 retrospective studies [50]. Beyond the comparability of complication rates and the shorter hospital stays of patients undergoing ablation, some authors propose that ablative techniques may also offer superior cost-effectiveness compared with surgical approaches [49, 51].

### Guidelines and future perspectives

Despite their good oncologic and clinical results, the classic thermal ablative techniques (RFA, MWA, and CA) are underrepresented in most guidelines, and recommendations do not adequately reflect the results (Table 2) [5–7]. For instance, the German National RCC guidelines recommend RFA and CA as options only for patients with renal cancers < 4 cm and with significant comorbidities, a limited life expectancy, or contraindications to general anesthesia, favoring surgery for these lesions whenever feasible [6]. There is no recommendation for T1b tumors [6]. In contrast, the European guideline of the European Association of Urology (EAU) takes a more favorable stance on ablative techniques, noting that comparative studies found no significant difference in overall complication rates between PN and percutaneous CA, while patients with lesions up to 4 cm treated with the percutaneous technique had a shorter average length of hospital stay [5, 52–54]. However, for T1b lesions, local tumor control rates drop significantly. On multivariable analysis, CA of T1b tumors was associated with a 2.5-fold increased risk of death from RCC compared with PN [5, 55]. Nevertheless, the panel concluded that the current data were inadequate to reach conclusions regarding the clinical effectiveness of CA vs PN. Given these uncertainties in the presence of only low-quality evidence, the guideline panel decided to recommend CA only for frail and/or comorbid patients with SRMs. Therefore, the recommendation is to offer surgery to achieve a cure for localized RCC [5]. While the EAU guidelines are similar to those of ESMO, both guidelines broaden the range of indications for patients with hereditary conditions, multiple or bilateral tumors, and impaired kidney function [5, 7]. The guidelines of the American Urological Association take a far more open position towards ablative procedures, more in line with currently available data and similar to the National Comprehensive Cancer Network (NCCN) guidelines [3, 8]. While the AUA guidelines state that clinicians should prioritize PN for SRM management when intervention is indicated clinicians should consider thermal ablation as an alternate approach in all patients with tumors < 3 cm in size, where oncologic outcomes are comparable for thermal ablation and PN [3]. It remains speculative whether the differences in recommendations may be attributable to the fact that, in the USA, both interventional radiologists and urologists perform thermal ablations. All guidelines concur on at least one aspect: a lesion suspected of being RCC must be histologically confirmed prior to or during image-guided ablation (Table 2). Studies like the EuRECA registry have already influenced the EAU guidelines to advocate for biopsies prior to IGA (rather than during IGA). However, no such guidance exists for PN, which skews oncological outcomes due to undiagnosed benign lesions (30% of SRM). [3, 5–7, 56]. Iezzi et al also report current proceedings from an international consensus meeting on ablation in urogenital diseases including ablation strategies in RCC [57].

## Conclusion

In conclusion, the use and incorporation of image-guided minimally invasive treatment options for RCC is on the rise. Existing data suggest that thermal ablation procedures (RFA, MWA, and CA) and PN yield comparable oncologic and clinical outcomes. Despite the data available, the recommendations for thermal ablations vary significantly across national and international guidelines, a discrepancy that will hopefully be corrected.

## Supplementary information

Electronic Supplementary Material

## Abbreviations

CA: Cryoablation
CSS: Cancer-specific
ESMO: European Society of Medical Oncology
HDR: High-dose-rate
IRE: Irreversible electroporation
LRFS: Local recurrence-free survival
MFS: Metastasis-free survival
MWA: Microwave ablation
NSS: Nephron-sparing surgery
OS: Overall survival
PN: Partial nephrectomy
RAPN: Robot-assisted partial nephrectomy
RCC: Renal cell carcinoma
RFA: Radiofrequency ablation
RPN: Robotic partial nephrectomy
SABR: Stereotactic ablative body radiotherapy
SRMs: Small renal masses
