# Supplementary material for: Image-guided percutaneous ablative treatments for renal cell carcinoma
Source: Eur Radiol. 2025 Mar 7;35(9):5324–36. doi: 10.1007/s00330-025-11480-w (PMC12350574; doi:10.1007/s00330-025-11480-w)
Supplement: Supplementary file 1 — Electronic Supplementary Material [file 330_2025_11480_MOESM1_ESM.pdf]

Image-guided percutaneous ablative treatments for renal cell carcinoma

Electronic Supplementary Material

Eur Radiol (2025) Auer TA, Uluk Y, Grasso RF et al.

Table S1: Comprehensive summary of the 25 most recent studies on hyperthermal ablation procedure including RFA and MW in RCC.

Author	No. of pats.	No. of tum.	Study type	Inclusion criteria	Age (years, Range)	Tumor Size (cm, Range)	Anesthesia	Guid-ance	Tech. success	Survival	Complications	FU (months)
Ruiz et al. (2024)	93	105	Retro.	T1 SRM <4cm	70 (34-87)	2.5 (1.0-4.2)	Sedation	CT	96%	n.a.	5% major c.	26
Radros et al. (2024)	435	453	Retro.	T1 SRM <4cm	73 (65-78)	2.1	General	US/CT	n.a.	n.a.	2% major c.	3
Iheanacho et al. (2024)	128	128	Retro.	T1 SRM <4cm	72 (64-78)	2.4 (1.5-3.4)	Sedation	CT	n.a.	n.a.	No major c.	16
Ruiz et al. (2024)	93	105	Retro.	T1 RCC up to 7cm	70 (34-87)	2.5 (1-4.2)	General	CT	96 %	n.a.	5% major c.	26
Chlorogiannis et al. (2023)	290	290	Retro.	T1 RCC up to 7cm	65 (54-77)	3.2 (1.9-4.5)	General	CT/MRI	n.a	MWA: 98%; RAPN: 100%	MWA: 16% major c.; RAPN: 8% major c.	77
Efthymiou et al. (2023)	76	76	Retro.	T1 RCC up to 7cm	70	3.0 (1.8-4.2)	Local	CT	T1a: 95%, T1b: 67%	n.a.	No major c.	58
Aurilio et al. (2023)	34	44	Retro.	T1 RCC up to 7cm	64	n.a.	General	US/CT	100%	Mean OS: 58 months	No major c.	58
Bertolotti et al. (2023)	82	90	Retro.	T1 RCC up to 7cm	69.6 (31-88)	2.3	Moderate sedation	US/CT	93%	n.a.	No major c.	26
Pandolfo et al. (2023)	789	789	Retro.	T1 SRM <4cm	69	2.55 (0.9-4.1)	n.a.	CT/MRI	100%	2Y RR: 2% (MWA)	3% major c.	26
Cazales et al. (2023)	150	150	Retro.	T1b RCC 4-7cm	76	4.6	General	US/CT/ MRI	88%	n.a.	No major c.	30
Aarts et al. (2023)	164	188	Retro.	T1 SRM <4cm	67 (59-73)	2.6 (2.0-3.2)	General	US/CT	RFA: 92%; MWA: 91%	n.a.	3% major c.	52 (RFA) 20 (MWA)
Grbanovic et al. (2023)	43	44	Retro.	T1 SRM <4cm	69 (52-81)	MWA: 2.4 (1.4-4), RFA: 2.6 (1.2-3.7)	General	CT	100%	n.a.	No mmajor c.	3
Rusinek et al. (2022)	140	140	Retro.	T1 RCC up to 7cm	79	2.8 (2.3 - 3.5)	Moderate sedation	US/CT/ MRI	n.a.	n.a.	No major c.	36

Lucignani et al. (2022)	166	166	Retro.	T1 SRM <4cm	73 (59-79)	2.45 (1.7-3.4)	Deep sedation	US/CT	9%	3Y RFS: 95% MWA;<br>3Y OSl: 88% MWA	5% major c.	26
Cheng et al. (2022)	166	175	Retro.	T1 SRM <4cm	n.a.	2.87 (0.6-7.0)	General	US	97%	(OS) 1Y, 3Y, 5Y were 100%, 93%, and 93%	No major c.	39
Luzzago et al. (2022)	432	432	Retro.	T1 RCC up to 7cm	67 (59-76)	2.5 (1.8-3.3)	General	US/CT	n.a.	n.a.	No major c.	22
Zhang et al. (2021)	8	20	Retro.	Bilateral RCC	59 (30-81)	2.74 (0.7-6.2)	General	US	100%	n.a.	No major c.	24
Meng et al. (2022)	15	16	Retro.	T1 SRM <4cm	59 (46-74)	2.3 (1.2-3.6)	Local	CT	100%	CSS: 100%; 1-, 2-, 3-Y OS: 100%, 93%; 93%	No major c.	24
Li et al.<br><br>(2021)	32	32	Retro.	T1 SRM <4cm	55 (39-67)	n.a.	General	MRI	100%	1-, 2-, and 3-Y (PFS): 97%; 94%; 84%	3% major c.	24
Chan et al. (2021)	296	296	Retro.	T1 RCC <7cm	68 (54 - 82)	2.72 (2.5 – 62.85)	General	CT/MRI	100	n.a.	CRYO: 15.25%;<br>RFA: 16.9%;<br>LPN: 10.9%	85
Bianchi et al. (2021)	816	816	Retro.	T1 SRM <4cm	65 (55-74)	3.0 (2.2-4.5)	Moderate sedation	US/CT	n.a.	5Y RFSR; RFA: 74%,	3% major c.	61
Guo et al. (2020)	10	14	Retro.	T1 SRM <4cm	68 (54-94)	3.9 (1.2-9.7)	General	CT	n.a.	n.a.	No major c.	6
De Cobelli et al. (2020)	72	83	Retro.	T1 SRM <4cm	70 (34-89)	2.2	Sedation	US/CT	94%	RR MWA: 3%	No major c.	22
Yong et al. (2020)	45	45	Retro.	T1 SRM <4cm	71 (31-87)	2.6	General	CT	100%	OS: 92%.	2% major c.	8
Guo et al. (2020)	23	23	Retro.	T1b RCC 4-7cm	74 (58-89)	5.2 (4.1-6.6)	General	CT	100%	OS 1Y: 95%, 2Y: 86%, and 3Y: 71%	No major c.	17
Andrews et al. (2020)	1798	1798	Retro.	T1 RCC up to 7cm	7	1.9	n.a.	CT/MRI	n.a.	5 Y CSS: RFA 96%	No major c.	n.a.

Table S2: Comprehensive summary of the 25 most recent studies on hypothermal ablation procedures / cryoablation in RCC.

Author	No. of pats.	No. of tum.	Study type	Inclusion criteria	Age (years, Range)	Tumor Size (cm, Range)	Anesthesia	Guid-ance	Tech. success	Survival	Complications	FU (months)
Allen et al. (2024)	207	207	Retro.	CA in RCC	65.8 (27-90)	3.01 (0.3-6.5)	General	CT	85%	CSS: 99% (5-year); OS: 78% (5-year)	7% major c.	12
Sun et al. (2024)	257	257	Retro.	T1a SRM <4cm	71 (40-92)	≤ 4	n.a.	US/CT	100%	n.a.	No major c.	12
Waidhauser et al. (2024)	72	72	Pros.	Early RCC no Mets	61 (42-76)	3.01 (2-4)	General	CT	100%	n.a.	No major c.	54
Abdelsam et al. (2024)	29	29	Retro.	T1a SRM <4cm	70 (0-81)	2.2 (1-4)	General	MRI	100%	5-year OS: 72%, 10-year OS: 56%	6.9% CD III	16
Pigg et al. (2024)	94	94	Retro.	Central RCC	68.2 (38-87)	3.7 (1.5-6.7)	n.a.	US/CT/MRI	97%	OS 94%, CSS 98%	6% major c.	32
Jensen et al. (2024)	56	56	Retro.	Endophytic SRM <4cm	61.5	2.6 (± 8.9 mm)	General	CT	100%	86% recurrence-free after one session	5% major c.	6
Yamagami et al. (2024)	27	27	Retro.	RCC <1cm	74 (37-90)	2.5 (1.2-3.7)	Moderate sedation	CT	100%	n.a.	No major c.	6
Neves et al. (2024)	200	200	Pros.	T1a SRM <4cm	63	2.6 (0.8-4.0)	General	CT	76%	n.a.	12% minor c.	76
Nowak et al. (2024)	6487	n.a.	Retro.	T1 RCC up to 7 cm	65.8	3.01 (0.3-6.5)	General	CT	n.a.	n.a.	No major c.	21
Raja et al. (2024)	100	100	Retro.	T1 RCC up to 7 cm	63 (32-90)	2.9 (0.6-6.2)	General	CT	98%	1-year CSS: 100%; 1-year OS: 94%	4% major c.	3
Pietersen et al. (2024)	70	70	Pros.	Solitary RCC	67.5 (61-75)	4.0 (1.5-7.0)	Local	CT	100%	n.a.	No major c.	6
Duus et al. (2023)	56	57	Pros.	T1 RCC up to 7 cm	66.5 (57-74)	3.3 (2.5-3.9)	General	US/CT	95%	n.a.	No major c.	26
Moulin et al. (2023)	25	26	Retro.	T1a RCC up to 7 cm	65	2.5	General	US/CT	100%	survival rate of 92%	No major c.	29
Barjlolle et al. (2023)	63	63	Retro.	T1b >4-7cm	79.5	4.5 (4.2-4.9)	General	CT	97%	3-year OS: 87%; 3-year CSS: 95%	13% (CIRSE grade 3) 1.6% Grade 5	n.a.

Filzah et al. (2023)	53	85	Pros.	T1 RCC up to 7 cm	52 (23-80)	2.5 (± 1.0)	General	CT/MRI	99%	5-year OS: 91%	1.7% major c.	30
Cazales et al. (2023)	150	150	Retro.	T1b >4-7cm	76.0	4.6	General	US/CT/MRI	99%	n.a.	No major c.	16
Nienke et al. (2023)	30	32	Retro.	RCC up to 5 cm	69 (38-83)	3.1 (1.6-5.1)	General anesthesia	MRI	n.a.	1-/2-year LTP: 90%;/ 77%	6.1% major c.	26
Bertolotti et al. (2023)	82	90	Retro.	T1 RCC up to 7 cm	69.6 (31-88)	2.1 (± 0.8)	Local with moderate sedation	US/CT	98%	n.a.	No major c.	43
Aikawa et al. (2023)	119	119	Retro.	T1b >4-7cm	80.0	4.5	General	CT/MRI	n.a.	5-year OS: 84% (PCA), 5-year CSS: 97%	3,4% major c.	53.
Pandolfo et al. (2023)	789	789	Retro.	T1a SRM <4cm	68.0	2.55 (0.9-4.1)	n.a.	CT/MRI	99%	2-year recurrence rate: 8%	2% major c.	18
Umakoshi et al. (2023)	9	9	Retro.	CKD 4/5 + TAE + CA in RCC	64.0	3.0 (1.7-4.7)	Local	Ct	100%	No local tumor progression occurred.	No major c.	58
Aurilio et al. (2023)	34	44	Retro.	RCC	64.0	n.a.	General	US/CT	100%	Mean (OS) was 58 (± 4.77) LTP: 7%	No major c.	3
Junker et al. (2023)	n.a.	165	Pros.	T1 RCC up to 7 cm	69.1	3.04 (± 0.93)	Sedation	CT	n.a.	n.a.	6% major c.	3
Junker et al. (2023)	n.a.	194	Pros.	T1 RCC up to 7 cm	69.4 (62.2-76.1)	3.1(1.5–5.8)	Sedation	CT	n.a.	n.a.	10% major c.	68
Gobara et al. (2022)	19	19	Pros.	RCC > 3 cm	75 (52-84)	3.9 (3.1–5.3)	n.a.	CT	n.a.	5-year OS: 95%; PFS: 84%; CSS: 100%	4x Grade 3 events	12
